# The role of ferroptosis in central nervous system damage diseases

**DOI:** 10.7717/peerj.16741

**Published:** 2024-01-30

**Authors:** Mingzhu Li, Shengbo Jin, Xudong Zhu, Jian Xu, Yang Cao, Haozhe Piao

**Affiliations:** 1 Department of Integrated Traditional Chinese and Western Medicine Medical Oncology, Cancer Hospital of China Medical University, Liaoning Cancer Hospital & Institute, Shenyang, Liaoning Province, China; 2 College of Acupuncture and Massage of Liaoning Chinese Traditional Medicine, Shenyang, Liaoning Province, China; 3 Department of General Surgery, Cancer Hospital of China Medical University, Liaoning Cancer Hospital & Institute, Shenyang, Liaoning Province, China; 4 Department of Colorectal Surgery, Cancer Hospital of China Medical University, Liaoning Cancer Hospital & Institute, Shenyang, Liaoning Province, China; 5 Department of Gynaecology, Cancer Hospital of China Medical University, Liaoning Cancer Hospital & Institute, Shenyang, Liaoning Province, China; 6 Department of Neurosurgery, Cancer Hospital of China Medical University, Liaoning Cancer Hospital & Institute, Shenyang, Liaoning Province, China

**Keywords:** Ferroptosis, Nerve damage, Redox, GPX4

## Abstract

Ferroptosis is a form of cell death, *i.e.*, programmed cell death characterized by lipid peroxidation and iron dependence, which has unique morphological and biochemical properties. This unique mode of cell death is driven by iron-dependent phospholipid peroxidation and regulated by multiple cell metabolic pathways, including redox homeostasis, iron metabolism, mitochondrial activity, and the metabolism of amino acids, lipids, and sugars. Many organ injuries and degenerative pathologies are caused by ferroptosis. Ferroptosis is closely related to central nervous system injury diseases and is currently an important topic of research globally. This research examined the relationships between ferroptosis and the occurrence and treatment of central nervous system injury diseases. Additionally, ferroptosis was assessed from the aspect of theory proposal, mechanism of action, and related signaling pathways per recent research. This review provides a relevant theoretical basis for further research on this theory, the prospect of its development, and the prevention and treatment of such diseases.

## Introduction

At present, programmed cell death (PCD) is regarded as the most important type of cell death (Bedoui, Herold & Strasser, 2020; Tower, 2015). Furthermore, PCD is a self-directed and ordered process that is crucial for the growth and maintenance of organisms (Kopeina & Zhivotovsky, 2022). PCD plays a crucial role in maintaining tissue and organ homeostasis by regulating the activation and expression of various fate-determining genes through complex and distinct signaling pathways (Lockshin, 2016). Moreover, recent genetic and biochemical studies have unveiled the remarkable flexibility and molecular plasticity within these PCD pathways, emphasizing their ability to adapt and maintain organismal homeostasis (Maniam & Maniam, 2021; Samir, Malireddi & Kanneganti, 2020). Besides the well-known apoptosis, PCD also includes autophagy, necroptosis, pyroptosis, ferroptosis, and other processes. They are morphologically similar to apoptosis, can also induce severe inflammatory responses, and significantly affect the homeostasis of the body (Li et al., 2023; Tong et al., 2022).

Among these PCDs, ferroptosis is a unique form of programmed cell death that is iron-dependent and characterized by the accumulation of lipid peroxides (reactive oxygen species (ROS). It is distinct from other types of cell death and is regulated by various factors including amino acids, lipids, redox, and iron metabolism (Dixon et al., 2012). The primary mechanism involves the catalysis of the highly expressed unsaturated fatty acids on the cell membrane to undergo lipid peroxidation under the action of divalent iron or ester oxygenase, thereby inducing cell death (Cao & Dixon, 2016). The decrease in the regulatory core enzyme (Glutathione peroxidase 4 (GPX4)) of the antioxidant system (glutathione system) is also associated with ferroptosis. The process is described as complex, but specific details of its intricacies remain unexplored (Dixon et al., 2014). Research has shown that ferroptosis is closely associated with the occurrence of a variety of diseases which also include central nervous system (CNS) injury diseases. The relationship between ferroptosis and the occurrence and treatment of such diseases, along with the research on ferroptosis in recent years, was analyzed based on theory proposal, mechanism of action, and related signaling pathways. Furthermore, growing research has increasingly demonstrated the significant regulatory roles of ferroptosis in the progression of CNS damage diseases (David et al., 2022; Jhelum et al., 2020; Xie et al., 2019; Zhu et al., 2022). Gaining a deeper understanding of the mechanism of ferroptosis may effectively improve the diagnosis and treatment of CNS diseases. Therefore, the aim of the review is to provide a relevant theoretical basis for further research on this theory, the likelihood of its development, and the prevention and treatment of CNS injury diseases.

## Survey Methodology

We searched related literature by pubmed using the key words (ferroptosis) AND (central nervous system damage)/(ferroptosis) AND (central nervous system disease)/(ferroptosis) AND (central nervous system injury). However, the papers which were not research articles or reviews were excluded.

### The concept of ferroptosis

Cell death is a key driver factor of degenerative diseases and also a fundamental characteristic of multicellular organism development. Many diseases can affect the rate of metabolism, which can lead to a reduction in the normal production of energy and biomolecules. For the normal development of multicellular organisms, cell death is very important, and in many diseases, it is abnormally initiated or inhibited. Up until the mid-20th century, cell death was believed to be uncontrollable before the idea of programmed cell death was introduced. The following decades led to the discovery of a type of programmed cell death called apoptosis. For the next few years, apoptosis was used interchangeably with programmed cell death; before the concept of programmed necrosis emerged in the early 21st century. This led to the discovery of a type of cell death that is regulated by their molecules, *i.e.,* “regulated cell death,” this term encompasses other types of cell death, such as necrosis and pyroptosis (Dixon et al., 2012; Santagostino et al., 2021; Tang et al., 2019).

In 2003, Erastin, a small molecule, was discovered to selectively attack tumor cells through a non-apoptotic mechanism (Dolma et al., 2003). In the following years, it was discovered that Erastin and several other compounds could activate a distinct form of cell death that relied on iron, and differed from apoptosis and other types of necrosis like pyroptosis (Yang & Stockwell, 2008). This iron-dependent cell death was termed “ferroptosis” in 2012 and is characterized by unique morphological, biochemical, and genetic features that set it apart from apoptosis, necrosis, and autophagy. Ferroptosis is closely associated with iron metabolism, lipids, and redox processes. Morphologically, it is characterized by mitochondrial atrophy, reduction or disappearance of mitochondrial cristae, increased density of mitochondrial membranes, and rupture of outer mitochondrial membranes. Biochemically, ferroptosis is marked by the accumulation of iron ions, aggregation of ROS, and lipid peroxidation (Dixon et al., 2012). Scientific literature has established a correlation between ferroptosis and various pathological features associated with tissue injury, as well as its physiological role in immune surveillance. Consequently, targeting ferroptosis inhibition holds promise as a potential therapeutic strategy for ferroptosis-related diseases. Notably, studies and reports have elucidated the induction of ferroptosis through catalyzing the elevated expression of unsaturated fatty acids on cell membranes, leading to lipid peroxidation under the influence of divalent iron or ester oxygenase. Additionally, investigations have explored alternative mechanisms, apart from GPX4 and glutathione (GSH) (Yang et al., 2014), employed by cells to counteract ferroptosis. Nonetheless, a comprehensive understanding of the intricate molecular pathways governing ferroptosis induction necessitates further research and analysis.

### Mechanism of ferroptosis

#### Relationships between ferroptosis and regulation of GPX4 and GSH lipid peroxidation

Ferroptosis is a form of cell death that was first reported in 2012 (Dixon et al., 2012). Initially, it was believed that the specific mechanism through which ferroptosis occurs was related to the metabolism of cysteine and glutathione and the peroxidation of phospholipids. Notably, ferroptosis was found to be characterized by the accumulation of lipid peroxides within certain cells. Inhibiting the defense system responsible for removing these lipid peroxides results in their accumulation to lethal levels. This can be achieved, for example, by inhibiting the cystine-glutamate reverse transporter protein, depleting the cellular antioxidant glutathione, reducing the glutathione-dependent GPX4 and ultimately leading to the accumulation of increased levels of lipid peroxides (Hirschhorn & Stockwell, 2019; Yang et al., 2014). Furthermore, the connection between ferroptosis and the role of GPX4 is evident in studies involving mice with a knockout *GPX4* gene. These mice exhibited neurodegenerative changes and cognitive impairment that were related to the activation of extracellular signal-regulated kinases, lipid peroxidation, and a significant increase in neuroinflammation (Dugger & Dickson, 2017). The research noted that the impact of the absence of GPX4 was quite severe.

**Figure 1:**
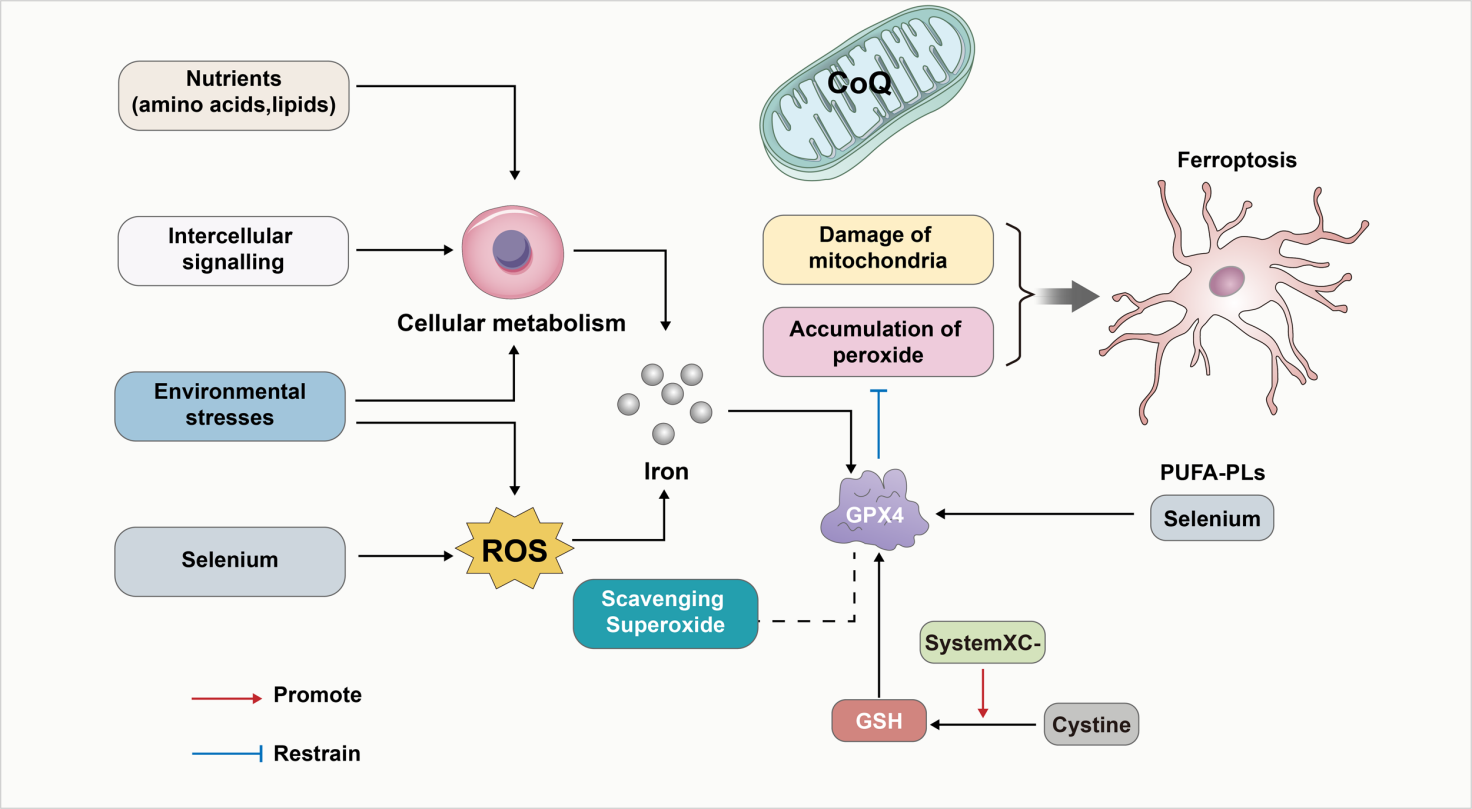
GPX4 as a key linker in the development of ferroptosis. GPX4 has been a key linker in the development of ferroptosis. First, GPX4 is an essential inhibitor of phospholipid peroxidation, together with the damage of mitochondria to contribute to the development of ferroptosis. In addition, the oxidized form of cysteine (Cystine) also counteracts ferroptosis by promoting the activity of GSH/GPX4. Furthermore, GPX4 induced ferroptosis can be influenced by environmental stress and intracellular and intercellular signal transport events which impact ROS levels, cellular metabolism and the level of Iron. In-depth studies have revealed that the deficiency of GPX4 can affect lipid peroxidation dependence. Also, Selenium/PUFA-PLs and scavenging superoxide affect the GPX4 induced ferroptosis. In addition, CoQ10 serves as a vital component of mitochondria and also inhibits lipid peroxidation outside the mitochondria. Consequently, the depletion of CoQ10 renders cells more susceptible to ferroptosis.

Various studies noted that reduced glutathione, selenium, and GPX4 contribute to the cellular antioxidant defense system and help regulate iron-dependent cell death. The most abundant reductant in mammalian cells, reduced GSH, functions as a cofactor for a variety of enzymes, including glutathione-s-transferases and glutathione peroxidases (GPXs). An important regulator of the occurrence of iron death is the trace element selenium, which is an essential for the biosynthesis of selenoproteins that clears the ROS. This also included GPX4 (Yang et al., 2014). It was noted that the oxidized form of cysteine (Cystine) also counteracts ferroptosis by promoting the activity of GPXs (Banjac et al., 2008; Mandal et al., 2010; Seiler et al., 2008; Shi et al., 2000; Yant et al., 2003), when absorbed by the cystine-glutamate reverse transporter (Bannai & Kitamura, 1980; Sato et al., 1999). Furthermore, ferroptosis can be influenced by environmental stress and intracellular and intercellular signal transport events which impact ROS levels and cellular metabolism. In-depth studies have revealed that the deficiency of GPX4 can lead to lipid peroxidation dependence. This in turn affects the neurodegeneration of hippocampal and cortical regions within the brain (Jia et al., 2020). Furthermore, the interplay of oxidative stress between cells can also affect ferroptosis, ultimately leading to nerve injury. The above process is also summarized in Fig. 1.

#### Hippo-YAP signaling pathway

**Figure 2:**
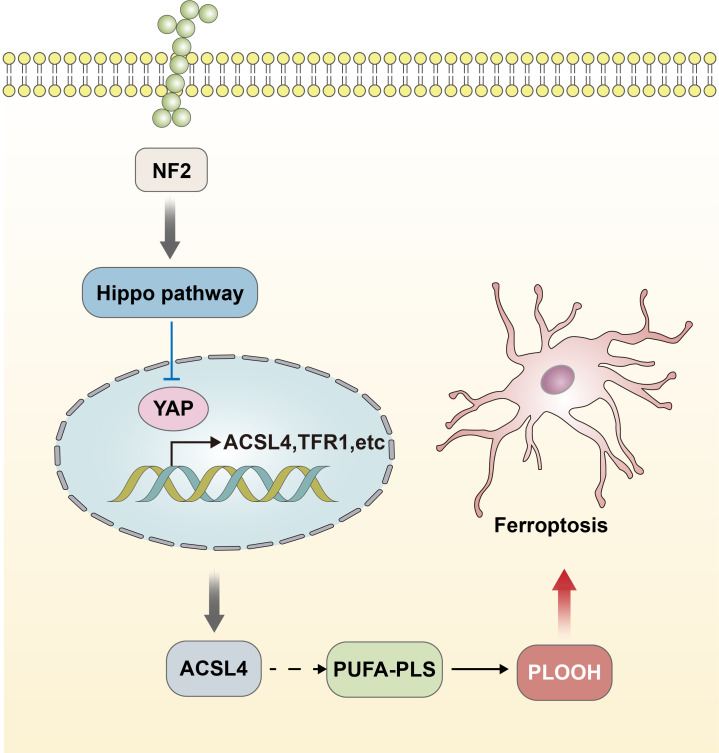
Hippo-YAP signaling pathway contributes to the occurrence of ferroptosis. The effect of the Hippo-YAP pathway on ferroptosis in epithelial cells is regulated by e-calmodulin-mediated cell–cell contact. This contact inhibits the expression of melin, leading to the activation of the Hippo signaling pathway through the *NF2* gene. Consequently, the YAP activity is suppressed. The Hippo-YAP pathway encompasses ACSL4, TFR1, and other possible genes that act as regulatory factors for ferroptosis. Notably, ASCL4 can further contribute to the activation of PUFA-PLs and PLOOH. The activity of the Hippo pathway performs a crucial function in the occurrence of ferroptosis, and heightened susceptibility to Hippo will increase the sensitivity of ferroptosis.

The functional performance of GPX4 is closely linked to the regulation of the Hippo-YAP signaling pathway (Wu et al., 2019; Zhang et al., 2022). The effect of the Hippo-YAP pathway on ferroptosis in epithelial cells is regulated by calmodulin-mediated cell–cell contact. This contact inhibits the expression of melin, leading to the activation of the Hippo signaling pathway through the Neurofibromatosis type 2 (NF2) gene. Consequently, the YAP activity, which is co-regulated by nuclear translocation and transcription, is suppressed. The Hippo-YAP pathway encompasses Acyl-CoA Synthetase Long Chain Family Member 4 (ACSL4), the transferrin receptor protein 1 (TFR1), and other possible genes that act as regulatory factors for ferroptosis. Notably, ACSL4 has been implicated as a transferrin receptor involved in sperm development. Furthermore, ASCL4 can further contribute to the activation of polyunsaturated fatty acid phospholipids (PUFA-PLs) and phospholipid hydroperoxides (PLOOH) (Schneider et al., 2009; Ufer et al., 2008). The activity of the Hippo pathway performs a crucial function in the occurrence of ferroptosis, and heightened susceptibility to Hippo will increase the sensitivity of ferroptosis (Fig. 2) (Wu et al., 2019). The data acquired indicated that the Hippo-YAP signaling pathway is intricately intertwined with ferroptosis and diseases caused by neurological damage. It was noted that this pathway is significantly upregulated and activated in the astrocytes of the optic nerve of mice with encephalomyelitis. This activation also resulted in activating both astrocytes and microglia, leading to the suppression of neuroinflammatory infiltration and the prevention of optic nerve demyelination (Wu et al., 2021). The involvement of the Hippo-YAP pathway in these processes highlights its potential in the modulation of ferroptosis-related pathways in the context of neurological damage.

### Ferroptosis-related inducers

#### Erastin and RSL3.

Erastin and RSL3 compounds were discovered initially as inducers for the occurrence of ferroptosis. The mechanism of both compounds for inducing ferroptosis involves targeting GPX4. Erastin and RSL3 can both directly lead to the inactivation of GPX4. In the case of Erastin, it can also indirectly lead to the inactivation of GPX4 by inhibiting the conversion of cystine, an essential component of glutathione synthesis. Consequently, lipid peroxidation is catalyzed, ultimately resulting not only in ferroptosis but also in neurodegenerative processes (Conteh et al., 2019).

#### FIN56.

The FIN56 compound is a novel chemical inducer of ferroptosis (Cao et al., 2019). It functions by a dual mechanism involving the depletion of mevalonate and GPX4 protein, as well as Coenzyme Q10 (CoQ10). CoQ10 serves as a vital component of mitochondria and also inhibits lipid peroxidation outside the mitochondria. Consequently, the depletion of CoQ10 renders cells more susceptible to ferroptosis. In addition, prevention of the activity of the HMG coenzyme A reductase further heightens the vulnerability of cells to ferroptosis (Cao et al., 2019; Shimada et al., 2016). This context was also presented in Fig. 1.

#### Lipoxygenases (LOXs).

Some studies have now found that LOXs compounds may also be inducers of ferroptosis (Chen et al., 2021; Yang et al., 2016). Ferroptosis can be inhibited by some LOXs pharmacological inhibitors (Li, Maher & Schubert, 1997), and baicalin can protect mice from an ischemic brain injury when *Alox15* is knocked out or when LOXs inhibitors are applied (van Leyen et al., 2006). However, some reports do not support this view. In the context of GPX4 knockout, further removal of the Alox15 gene did not prevent ferroptosis of fibroblasts in mice, nor did it prevent acute ischemic kidney injury and was linked to *in vivo* lethality (Friedmann Angeli et al., 2014). However, it is noteworthy that LOXs are primarily implicated in ferroptosis triggered by cysteine deficiency, as opposed to ferroptosis triggered by GPX4 deficiency. This observation suggests the necessity of a distinct model to further analyze the role of LOXs in diseases related to ferroptosis. The data indicates the possibility of an alternate mechanism compensating for the absence of ALOX15 activity and suggests that LOXs may be involved in certain cases of ferroptosis-related diseases. It is also worth considering that the inhibitor typically employed to specifically LOXs may possess additional properties beyond their intended specificity. These might exhibit non-specific activity as free radical trapping antioxidants, leading to their inhibitory effects on ferroptosis through this mechanism (Zilka et al., 2017). Certainly, the predominantly used LOX inhibitors exhibit antioxidant activity, enabling them to capture free radicals. This poses a challenge to the conventional understanding of the role of LOXs in diseases associated with ferroptosis. Furthermore, it was noted that the occurrence of RSL3-induced ferroptosis was not prevented by the combined downregulation of all LOX isozymes. Interestingly, this downregulation had a significant rescuing effect on ferroptosis induced by Erastin, as it is possible that Erastin treatment is associated with the activation of LOX enzymes (Shah, Shchepinov & Pratt, 2018; Yang et al., 2016). These findings suggest that while oxidation may not be the primary factor driving ferroptosis in most cases, it could still contribute to the initiation and progression of injury under certain circumstances. Accordingly, in specific mouse models of cancer inhibition or neurodegeneration, the deficiency of Alox12 or Alox15 showed a positive effect (Chu et al., 2019). A summary of the inducers of ferroptosis is shown in Table 1.

**Table 1:** A summary of the inducers of ferroptosis.

**Name**	**Function**	**Molecular mechanism**
Erastin	Inducer of ferroptosis	Directly lead to the inactivation of GPX4; indirectly lead to the inactivation of GPX4 by inhibiting the conversion of cystine
RSL3	Inducer of ferroptosis	Directly lead to the inactivation of GPX4
FIN56	A novel chemical inducer of ferroptosis	Depletion of mevalonate and GPX4 protein, as well as Coenzyme Q10
LOXs	Inducer of ferroptosis	Deficiency of cysteine

### Iron metabolism is involved in the regulation of ferroptosis

The role and regulation of iron in ferroptosis have recently been reported in studies despite the inclusion of “ferro” (meaning iron) in the name of this form of cell death. At the outset, understanding the precise cause of lipid peroxidation, a critical characteristic of ferroptosis, presented a challenge. It remained uncertain whether the transfer of an unstable iron pool within the cell membrane and its subsequent reaction with lipid peroxides was the primary trigger, or if the peroxidation process was predominantly driven by the activity of iron-dependent enzymes alone. Recent studies have shown that iron death is triggered by iron-dependent lipoxygenases by generating lipid peroxides. Additionally, it has been observed that unstable iron, which is not bound to these enzymes, further facilitates the catalysis of these peroxides, resulting in increased lipid peroxidation (Shah, Shchepinov & Pratt, 2018; Wenzel et al., 2017). In ferroptosis, cytochrome P450 oxidoreductase, an iron-dependent enzyme has been implicated in the process of lipid peroxidation, potentially playing a significant role (Zou et al., 2020). A critical determinant of ferroptosis progression is the abundance of ferritin, a protein involved in iron storage. Higher levels of ferritin confer resistance against ferroptosis, as they sequester iron and limit the availability of unstable iron pools. Conversely, the depletion of ferritin may result in the release of iron into these unstable iron pool, leading to greater sensitivity against ferroptosis. Prior experiments have demonstrated that ferritin-targeted autophagy, known as ferritinophagy, triggers the breakdown of ferritin within lysosomes and releases iron into unstable iron pools, thereby increasing the sensitivity to ferroptosis (Gao et al., 2016; Hou et al., 2016). Furthermore, Prominin2 (PROM2) inhibits ferroptosis by promoting iron transport out of the cell by multivesicular bodies and exosomes containing ferritin (Brown et al., 2019).

### The role of mitochondria in ferroptosis

Mitochondria generates key senescence metabolites during cellular metabolism and alters the sensitivity of cells to the tricarboxylic acid (TCA) cycle. Thereby increasing ferroptosis by altering the unstable iron content of cells. Mitochondria play a crucial role in regulating the respiration rate, and an increase in their activity can lead to enhanced production of ROS by automatically increasing the availability of cellular iron (Gao et al., 2015). Therefore, the normal degradation of ferritin within mitochondria along with its metabolic function appears to promote ferroptosis (Gao et al., 2016; Hou et al., 2016). Similarly, transferrin and its receptor play a collaborative role in iron homeostasis, facilitating the import of iron into cells. Moreover, it has been established that they actively participate in enhancing the export of iron from cells to promote oxidation (Hou et al., 2016; Tuo et al., 2017), thus promoting the formation ferroptosis. Additionally, recent studies have proposed a connection between the RSL3 induced-inhibition of O-GlcNAcylation modification and the increased autophagy of both iron and mitochondria. This enhanced autophagy regulation influences the sensitivity to ferroptosis, leading to the accumulation of unstable iron and facilitating the progression of ferroptosis (Yu et al., 2022). In nerve cells, the contraction of mitochondria and the accumulation of iron increase the occurrence of ferroptosis and lead to increased nerve injury, such as lumbosacral nerve root avulsion injuries (Zhou et al., 2022).

### The relationship between ferroptosis and central nervous system injury diseases

Many studies have reported that ferroptosis plays an important role in the progression of central nervous system injury disorders. These include stroke (Cui et al., 2021; Liu et al., 2020), Parkinson’s disease (Mahoney-Sanchez et al., 2021), Alzheimer’s disease (AD) (Yan & Zhang, 2019), and Huntington’s traumatic brain injury (TBI) (Geng et al., 2021; Mi et al., 2019). A growing number of studies have shown that ferroptosis plays a crucial role in neurodegenerative diseases, which are characterized by the accumulation of Fe^2+^, lipid peroxidation, and alterations of mitochondrial structure.

### Stroke

Current studies suggest that ferroptosis is an important causative element of cerebral ischemia/reperfusion (I/R) injury. Research indicates that spermidine/spermine n1-acetyltransferase 1 (SSAT1) activates the upregulation of arachidonic acid 15-lipoxygenase (ALOX15). Consequently, the upregulation of ALOX15 resulted in the triggering of ferroptosis in brain neurons, thereby exacerbating brain ischemia/reperfusion injury (Zhao et al., 2022). This form of cell death has also been implicated in the pathophysiological process of TBI. Furthermore, studies have shown that melatonin can inhibit ferroptosis in the brain neurons after TBI and thus have a neuroprotective effect on the brain (Rui et al., 2021).

### AD

AD is characterized by the progressive occurrence of cortical and hippocampal neuronal dysfunction and death. Its main pathological changes include neuronal degeneration and synaptic loss (Hansson, 2021), with neuronal death as a potential cause of AD (Hambright et al., 2017). Ferroptosis is characterized by increased iron content and altered lipid peroxidation in AD along with cognitive impairment (Chen et al., 2015; Hambright et al., 2017). Increased intracellular iron content directly induces oxidation-related neuronal damage (Thirupathi & Chang, 2019; Yoo et al., 2010). Thereby, the oxidation-related neuronal damage further promoted the generation and increase of ROS (Derry et al., 2020), which may be involved in the ferroptosis pathway (Li et al., 2019). The occurrence of mitochondrial oxidative dysfunction, reduced DNA repair, apoptosis, and increased autophagy associated with this leads to neuronal loss and an increased risk of AD (Brickman et al., 2021; Goel et al., 2022).

### Parkinson’s disease

Parkinson’s disease is characterized by accumulation of misfolded alpha synuclein and loss of neurons in several brain regions, particularly dopaminergic neurons in the substantia nigra (Guiney et al., 2017). The key therapeutic goal is to improve dopamine levels in the brain and prevent neuronal degeneration. Notably, dopaminergic neurons in Parkinson’s pathology are enriched in iron, which plays a role in both enzymatic and non-enzymatic aspects of dopamine metabolism (Moreau et al., 2018). Ferroptosis associated with iron content is a key cell death pathway in dopaminergic neurons (Do Van et al., 2016). Iron induces the oxidation of dopamine and the aggregation of a-synuclein. Moreover, excess free iron can reduce the activity of GPX4, leading to the depletion of GSH (Duce et al., 2017). Subsequently, the reduced ability of cells to scavenge ROS leads to an excess of ROS and induces the accumulation of lipid peroxidation and iron-dependent free radicals on neuronal and astrocyte membranes (Angelova et al., 2015). This ultimately results in ferroptosis, which in turn leads to increased loss of dopaminergic neurons in Parkinson’s disease, and is a possible potential pathogenic mechanism (Imai et al., 2017; Stockwell et al., 2017).

### Neuropathic pain due to spinal cord injury

In chronic neuropathic pain (NP), the level of Acyl CoA synthetase long-chain family member 4 (ACSl4) increased due to reduced levels of GPX4 (Guo et al., 2021; Wang et al., 2021). This led to an increase in the number of mitochondria and a decrease in the average mitochondrial area, along with elevated iron ion levels in the spinal cord of rats. The increase in ACS14 induces ferroptosis and further activates neurons in the dorsal horn of the spinal cord and astrocytes involved in the maintenance of chronic pain.

Experimental studies have shown that the spinal cord segments of rats can upregulate the expression of GPX4 and downregulate the expression of ACSL4 when treated with the ferroptosis inhibitor ferritin. Nonetheless, the opposite effect is achieved when treated with the ferroptosis inducer Erastin (10 mg/kg). Iron ion levels are elevated in the spinal cord during NP and can be modulated by interventions such as Cci-induced iron statin-1 and Erastin; decreased by the former and increased by the latter. Ferroptosis plays a role in the development of NP in rats following peripheral nerve injury by impairing the activation of neurons and astrocytes in the spinal dorsal horn. Inhibition of ferroptosis with lipostatin-1 significantly alleviates mechanical and thermal hypersensitivity associated with NP (Guo et al., 2021).

### Summary and outlook

Since the term ferroptosis was coined in 2012, the study of ferroptosis has exploded over the past few years. It is a regulated iron-dependent programmed cell death form characterized by the accumulation of lipid peroxidation. It has also been linked with ROS, mitochondrial metabolism, and iron metabolism; however, the exact molecular mechanism remains unelucidated. Current studies have found that the process may be correlated with the Hippo-YAP signaling pathway, but the exact mechanisms and the possible molecular mechanism markers are still being explored. Tan, Fang & Gu (2021) summarized a review about the role of ferroptosis in central nervous system diseases. Their review primarily dealt with lipid metabolism, iron metabolism, the GSH-dependent pathway and the CoQ10-dependent pathway. However, in our review, we mainly summarized and discussed GPX4 and GSH lipid peroxidation, the Hippo-YAP signaling pathway, ferroptosis-related inducers, and mitochondria in the process of ferroptosis. Notably, the role of ferroptosis-related inducers was explored concerning the regulation of ferroptosis. These points are the main differences between these two reviews, and we believe our review is more accessible to a different audience compared with the review of Tan, Fang & Gu (2021).

Furthermore, during the development of nerve injury diseases, nerve cell oxidative stress, mitochondrial damage, and lipid oxidation are closely related to the occurrence of ferroptosis in nerve cells (Yang & Stockwell, 2016). Studies related to central nerve injury diseases have implicated ferroptosis in the development of ischemic brain nerve injury, traumatic brain nerve injury and neurodegenerative brain diseases. In the context of peripheral nerve injury disease, ferroptosis plays an important role in neuropathic pain linked with nerve injury, which is closely related to pain sensitization. The use of ferroptosis inhibitors has shown promise for providing a neuroprotective effect by reducing the onset of neuropathic pain.

It has been established that oxidative stress and mitochondrial dysfunction are involved in the occurrence and development of many neurodegenerative diseases, such as AD and Parkinson’s disease (Dell’Orco et al., 2016; Jackson, 2009). GSH, an important antioxidant in the body, can scavenge ROS, reduce hydrogen peroxide to H_2_O, repair biofilms and resist ferroptosis. In addition, the depletion of GSH can also induce a decrease in the activity of GPX4, which is a central regulatory factor of ferroptosis and antioxidant reactions. GPX4 abnormalities are thought to be a necessary precondition for cell ferroptosis (Jia et al., 2020), and can contribute to neurodegeneration (Cao et al., 2019).

Under oxidative stress conditions, the level of ROS in nerve cells increases, and promotes the peroxidation of intracellular membrane lipids and proteins, which in turn leads to neuronal oxidative injury (Dixon & Stockwell, 2014). Furthermore, it has been documented that excessive oxidative stress is one of the main predispositions for neurological impairment. Ferroptosis inhibitors, including some ROS scavengers, can effectively block the generation of iron-catalyzed ROS, resist oxidative stress reaction, clear intracellular accumulation of ROS, and prevent ferroptosis in cells. ROS scavengers are also commonly used as anti-nerve injury drugs. However, further investigation is needed to determine the precise mechanisms underlying their neuroprotective effects, whether it be through scavenging antioxidant stress or by blocking ferroptosis.

Several types of ferroptosis inhibitors have been found, and preclinical trials have exhibited their efficacy in reducing the occurrence of ferroptosis in neuronal cells, which may mitigate the associated nerve injury diseases in a variety of central nervous system diseases consequently. Clinical trials are underway for the treatment of Parkinson’s disease by using related inhibitors. Following the dose of 15 mg/Kg body weight, patients with Parkinson’s disease received oral deferiprone (DFP) for 36 weeks. As a result, the nigrostriatal iron context significantly decreased in the DFP group. Although other results were not very promising (Devos et al., 2022). However, the clinical application of ferroptosis inhibitors for other diseases are yet to be explored. In conclusion, further research is needed to investigate the mechanisms of ferroptosis associated with nerve injury. It is essential to explore drugs and new drug targets for the prevention and treatment of ferroptosis associated with nerve injury.

## Additional Information and Declarations

### Data Availability

The following information was supplied regarding data availability: This is a literature review.
